# The genome of *Alcaligenes aquatilis* strain BU33N: Insights into hydrocarbon degradation capacity

**DOI:** 10.1371/journal.pone.0221574

**Published:** 2019-09-24

**Authors:** Mouna Mahjoubi, Habibu Aliyu, Simone Cappello, Mohamed Naifer, Yasmine Souissi, Don A. Cowan, Ameur Cherif

**Affiliations:** 1 Univ. Manouba, ISBST, BVBGR-LR11ES31, Biotechpole SidiThabet, Ariana, Tunisia; 2 Institute of Process Engineering in Life Science 2: Technical Biology, Karlsruhe Institute of Technology, Karlsruhe, Germany; 3 Istituto per l’Ambiente Marino Costiero (IAMC)-CNR of Messina. Sp. San Raineri, Messina, Italy; 4 Centre for Microbial Ecology and Genomics, University of Pretoria, Pretoria, South Africa; Universidade de Coimbra, PORTUGAL

## Abstract

Environmental contamination with hydrocarbons though natural and anthropogenic activities is a serious threat to biodiversity and human health. Microbial bioremediation is considered as the effective means of treating such contamination. This study describes a biosurfactant producing bacterium capable of utilizing crude oil and various hydrocarbons as the sole carbon source. Strain BU33N was isolated from hydrocarbon polluted sediments from the Bizerte coast (northern Tunisia) and was identified as *Alcaligenes aquatilis* on the basis of 16S rRNA gene sequence analysis. When grown on crude oil and phenanthrene as sole carbon and energy sources, isolate BU33N was able to degrade ~86%, ~56% and 70% of TERHc, *n*-alkanes and phenanthrene, respectively. The draft genome sequence of the *A*. *aquatilis* strain BU33N was assembled into one scaffold of 3,838,299 bp (G+C content of 56.1%). Annotation of the BU33N genome resulted in 3,506 protein-coding genes and 56 rRNA genes. A large repertoire of genes related to the metabolism of aromatic compounds including genes encoding enzymes involved in the complete degradation of benzoate were identified. Also genes associated with resistance to heavy metals such as copper tolerance and cobalt-zinc-cadmium resistance were identified in BU33N. This work provides insight into the genomic basis of biodegradation capabilities and bioremediation/detoxification potential of *A*. *aquatilis* BU33N.

## Introduction

Petroleum compounds are ubiquitous environmental pollutants with potentially harmful impacts on human and ecological health balance [1]. Physico-chemical treatments, used to remove hydrocarbons from contaminated sites, are extremely expensive, give rise to more toxic compounds and have limited effectiveness leading to modification and destruction of biological materials [2]. Therefore, there is an urgent need to mitigate pollution and promising biological strategy for the decontamination of hydrocarbon polluted sites has been carried out based on the application of obligate hydrocarbonoclastic bacteria, OHCB [3–5]. Moreover, the identification and characterization of novel hydrocarbon degrading bacteria is still essential to enhance and reach efficient bioremediation treatments.

Several microorganisms/bacteria are currently used as bioremediation agents, including isolates of the genera *Marinobacter*, *Thallassolituus*, *Cycloclasticus* and *Oleispira* [5, 6].

*Alcaligenes* is a genus of Gram-negative bacteria isolated from a wide variety of environments, including water, soil and clinical samples [7, 8]. The genus was proposed in 1919 and classified in the family *Alcaligenaceae* (Betaproteobacteria class) with the type species identified as *Alcaligenes faecalis* now comprising three subspecies; *A*. *faecalis* subsp. *Faecalis*, *A*. *faecalis* subsp. *parafaecalis* and *A*. *faecalis* subsp. *phenolicus* [9–11]. In addition, three other species, namely, *A*. *aquatilis* [12], *A*. *endophyticus* [13] and *A*. *pakistanensis* [14] have been circumscribed in the genus. [12, 14].

Potential applications of members of the genus in agriculture and pharmaceutical industries, and the ability of A. *faecalis* to degrade pesticides have been reported [15, 16]. *Alcaligenes eutrophus* A5 was reported to degrade the pesticide DDT [17]. *Alcalignes aquatilis* LMG 22996^T^ was first isolated from sediments of the Weser Estuary, Germany, and from a salt marsh on Shem Creek in Charleston Harbor, USA [12]. *A*. *aquatilis* F8 strain has recently been reported as a cationic biosurfactant producer (CBS) [18]. *Alcaligenes aquatilis* QD168 (CCUG 69566), a marine hydrocarbon-degrading bacterium, was recently isolated from a crude oil-polluted marine sediment sample from Quintero Bay, Central Chile exhibiting a potential adaptation to environmental stressors such as toxic compounds, high salinity, and oxidative stress [19, 20]. These characteristics suggest the potential of the strain in bioremediation of oil-contaminated sites.

In this work, we report the characterization and genome sequencing of an oil-degrading bacterium *Alcaligenes aquatilis* (strain BU33N) isolated from hydrocarbon polluted sediments located at the refinery harbor of the Bizerte coast, North of Tunisia. To the best of our knowledge, this paper provides the first detailed description of the ability of *Alcaligenes aquatilis* to use and degrade hydrocarbons based on cultivation experiments and genomic analysis. Knowledge of this genomic information may facilitate the efficient use of this strain for biotechnological and bioremediation applications.

## Materials and methods

### Strain isolation

Strain BU33N was isolated from hydrocarbon polluted marine sediments located at the refinery harbor of the Bizerte coast, northern Tunisia (37°16'8"N, +9°53'19"E). The strain was isolated on mineral medium ONR7a supplemented with 1% crude oil as the sole carbon source at 30°C.

### Culture conditions

Strain BU33N was tested for its ability to utilize various hydrocarbons (pristane, phenanthrene, pyrene, naphtalene, carbazole, octadecane, fluoranthene, dibenzothiophen, dibenzofuran, squalene, anthracene and xylene) as a sole carbon and energy source. BU33N was inoculated on ONR7a agar media containing specified amounts of hydrocarbons and incubated for 7 days at 30±1°C. Growth of the bacterial colonies was considered as positive result of degradation.

### Hydrocarbon degradation analysis

For hydrocarbon degradation analysis, strain BU33N was cultured in ONR7a liquid mineral media [21] supplemented with 5 g L^-1^ Na-acetate for 48 h at 28±1°C. Cells were collected via centrifugation (10 min, 14.000 g) and washed twice in phosphate buffered saline (PBS 1×; 140 mM NaCl, 2.7 mM KCl, 4.3 mM Na_2_HPO_4_.7 H_2_O and 1.5 mM KH_2_PO_4_). Bacterial inocula (~10^6^ cells ml^-1^ measured by the DAPI count method) were added to 50 mL ONR7a liquid mineral medium supplemented with sterile Arabian Light Crude Oil (1%, v/v), pristane (1% v/v) and 50 ppm (final concentration) of phenantrene (PHE, C_14_H_10_, Sigma Aldrich, Milano–Italy). Cultures containing the same amount of hydrocarbons but without bacterial inoculation were used as abiotic controls. Cultures were incubated at 28± 1°C for 21 days with shaking (Certomat IS B. BraunBiothec International, 100 ×*g*) [21].

Qualitative and quantitative analysis of hydrocarbons [total extracted and resolved hydrocarbons and their derivatives (TERHCs) and phenanthrene derivatives] were carried out using a Master GC DANI Instruments GC-FID (Development ANalytical Instruments DANI Instruments S.p.A., Milan, Italy), equipped with SSL injector and FID detection. Hydrocarbons were extracted following the 3550C EPA (Environmental Protection Agency) procedure as previously reported [22]. The amount of biodegradation was expressed as the percentage of hydrocarbon degraded compared to abiotic control.

### Screening of biosurfactant production and emulsification activity

Biosurfactant production was screened by hemolytic activity and the Blue agar plate method [23, 24].The hemolytic activity assay was carried out by streaking strain BU33N on blood agar plates containing (5%, v/v) sheep blood and incubated for 48 h at 30±1°C. The plates were visually inspected for clearance zones around the colonies, as an indication of biosurfactant production. The CTAB (Cetyl trimethylammonium bromide).agar plate method [23] was used for detection of extracellular surfactant. BU33N was inoculated on solid ONR7a medium with cetyltrimethyl ammonium bromide (0,5 mg /ml) and methylene blue (0,2 mg /ml), supplemented with pyruvate as carbon source, productive colonies were indicated by the presence of dark blue halos [23].

BU33N cultures was prepared in Tryptic Soy Broth (TSB) for 48 h at 28±1°C until OD_600_ approx. 0.5). Cultures were centrifuged (10 min 14.000 g). Emulsification activity (E24) was determined by the addition of 3 ml of culture supernatant to 3 ml of crude oil. The mixture was vortexed at high speed for 3 min. After 24 h, emulsification activity was estimated as the height of the emulsion layer divided by the total height, expressed as a percentage [25].

### Morphological, phenotypic and molecular characterization of strain BU33N

Scanning electron microscopy was used to visualize the cellular morphology of BU33N strain. Cultures of BU33N were centrifuged (10 min 14.000 g) and bacterial biomass was fixed in 2.5% gluteraldehyde in 0.075 M K-phosphate buffer (pH 7.4) for 2 h at room temperature. The preparation of the Scanning Electron Microscope (SEM) was performed according to Stanton et al. [26], and samples visualized using a JCM-5700 Scanning Electron Microscope, resolution 0.6nm, specimen size 5 mm ∅ × 0.6 mm high, with a Gatan Digital Micrograph imaging system and SE & BS detectors.

Growth of BU33N strain at different pH values (5–11) using hydrochloric acid HCL (1M) and sodium hydroxide NaOH (1M) for pH adjustment, temperatures (25, 30, 40, 45, 50°C) and salinity values (0, 5, 10, 15, 25, 30% w/v NaCl) was determined in liquid TSB media. Culture OD values were measured after 3 days of incubation at 30°C.

For DNA extraction, strain BU33N was grown aerobically on TSB; pH 7.6±0.1 at 28±1°C).

### Strain BU33N genome sequencing

DNA isolation was carried out on mid-log phase cells by sodium dodecyl sulfate (SDS)-proteinase K treatment with an additional equal volume of CI (chlorophorm/isoamyl alcohol 24:1 v/ v) [27]. Purified genomic DNA was sequenced on an Illumina MiSeq platform (MRDNA, USA). The 9,357,646 paired reads were filtered according to read quality, and reads below a mean quality score of 23 were removed using prinseq-lite software. The reads were assembled using SPAdes [28]. The 16S rRNA gene was identified in the assembled genome using RNAmmer [29] and identified using EzBioCloud [30]. Similarly, RNAmmer [29] was used to predict the 16s rRNA genes of recently published genomic relative of BU33N, *A*. *aquatilis* strain QD168 (GenBank: CP032153.1). The genome predicted 16 rRNA genes and those of all type strains of the genus *Alcaligenes* were aligned and trimmed using MAFFT [31] and trimAl [32], respectively. A maximum likelihood (ML) tree was constructed based on TN+F+G4 model and the tree topology was evaluated by performing bootstrap analysis of 1000 data sets using IQ-TREE [32]. Furthermore, the genomic relatedness between BU33N and QD168 was assessed using GGDC 2.1 [33] and OrthoANI [34]. The genome of strain BU33N was annotated using RAST pipeline [35].

### Comparative genome analysis

CGView Server [36] was used for circular representation of multiple genomes. The draft genome of strain BU33N was used as the reference genome and was compared with genomes of *Alcaligenes faecalis* subsp. *phenolicus* (DSM16503), *Alcaligenes faecalis* ZD02 (CP013119.1) and *Alcaligenes aquatilis* QD168 (CCUG 69566). For the purpose of analyzing aromatic compound degradation pathways in BU33N, selected gene clusters linked to biodegradation of benzoate were compared with those from four hydrocarbon degrading bacteria. Selection of the genomic regions was based on annotations obtained using RAST subsystems. The genomic regions were extracted and structurally annotated with Prokka [37] by uploading the sequences to the Galaxy web platform, using the public server at usegalaxy.org [38]. Easyfig [39] was used to compare and visualize the gene clusters.

### Nucleotide sequence accession numbers

The *Alcaligenes aquatilis* BU33N genome sequence is deposited in the Bioproject Genomes online database with id PRJNA386470. The complete genome sequence was deposited in Genbank under accession number CP022390. The version described in this paper is version CP022390.

## Results and discussion

### Characterization of strain BU33N

**Fig 1:**
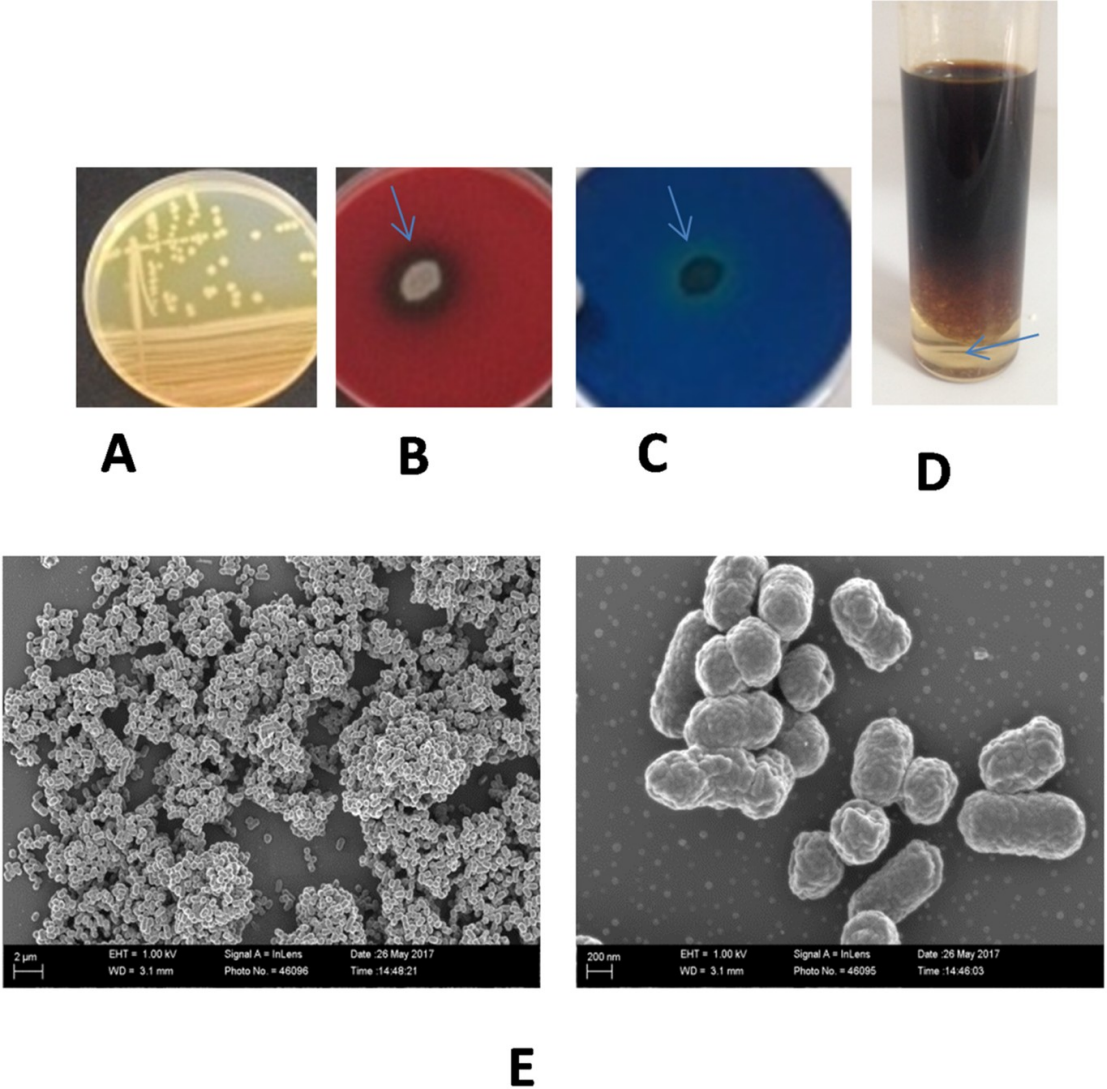
Characterization of *Alcaligenes aquatilis* BU33N. **A**) Colonies of BU33N on TSA medium **B**) Biosurfactant production (hemolytic activity); **C**) Biosurfactant production [Blue agar (CTAB) method]; **D**) emulsification activity (E24 test), **E)** Scanning electron micrographs of strain BU33N grown on TSA medium (at 28±1°C for 48h).

Isolation of BU33N was achieved using enrichment cultures using ONR7a medium supplemented with sterile crude oil (1% v/v) as the sole carbon and energy source [25]. The BU33N isolated was selected due to its rapid growth in liquid media with crude oil as sole carbon and energy source. BU33N cells were short rods, and colonies grown on TSA plates after 48h of incubation at 28°C were circular, yellow-pigmented and low-convex in form (**Fig 1, Table 1**). The strain grew in a temperature range between 20 and 40°C (optimal growth at 28°C) and at pH range from 5.5 to 10.0 (optimal growth at pH 7.5). BU33N showed production of putative biosurfactants (hemolytic assay and CTAB method). The latter method is considered to specifically indicate the production of glycolipid biosurfactants [23]. BU33N showed a clear emulsification activity (>25%) **(Fig 1).**

**Table 1:** Classification and general features of *Alcaligenes aquatilis BU33N*.

Property	Term	Type strain
Classification	Domain–*Bacteria*	LMG 22996^T^ [12]
	Phylum–*Proteobacteria*	
	Class–*Betaproteobacteria*	
	Order–*Burkholderiales*	
	Family–*Alcaligenaceae*	
	Genus–*Alcaligenes*	
	Species–*Aquatilis*	
	Strain BU33N	
Gram stain	Gram negative	LMG 22996^T^
Cell shape	Short rods	
Motility	Motile	LMG 22996^T^
Temperature range	20–40°C	
Optimum temperature	28°C	
pH range	5,5–7,5	
pH, optimum	7	
Carbon source	glucose, sucrose, sodium acetate, Tween-80 crude oil, phenanthrene, pyrene, carbazole	
Energy source	Heterotrophic	
Habitat	Hydrocarbon contaminated sites	
Salinity	0–30%	
Oxygen requirement	Strictly aerobic	LMG 22996^T^
Geographic location	Tunisia (37°16'8"N, +9°53'19"E).	
Date collection	September 2013	
Depth	10 cm	

Partial 16S rRNA gene sequencing of strain BU33N showed high level of identity of 99.91% with *Alcaligenes aquatilis* LMG 22996^T^ (AJ937889.1). Subsequently, full length 16S rRNA gene was predicted from the assembled genome using RNAmmer. The 16S rRNA gene sequence showed highest similarity (99.91%) to that of *Alcaligenes aquatilis* LMG 22996^T^. However, the BU33N 16S rRNA gene shares 100% identity with one of the three copies of the gene predicted on the complete genome of QD168. The other two genes were 99.74 and 99.87 identical to the BU33N 16S rRNA gene **(S1 Table).**

**Fig 2:**
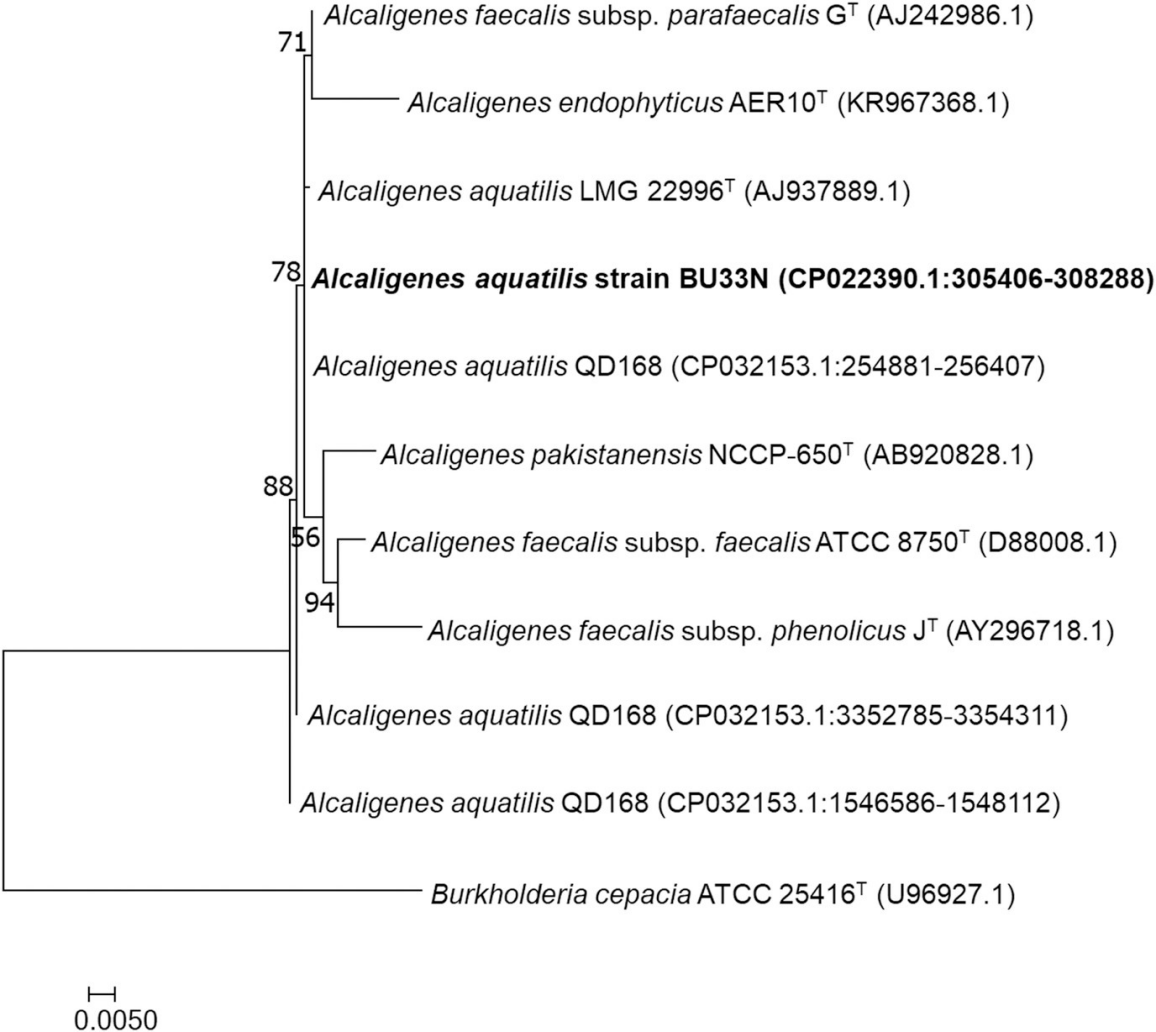
Phylogenetic analysis of 16S rRNA gene sequence of bacterial isolate *Alcaligenes aquatilis* strain BU33N. The maximum likelihood (ML) tree was generated based on the alignment of the 16s RNA genes of BU33N, QD168 and the type species of the genus *Alcaligenes*. The ML was constructed based on the TN+F+G4 model using IQ-TREE with confidence values based on 1000 bootstrap replicates. *Burkholderia cepacia* was used to position the root of the tree.

A phylogenetic tree showing the relationship of *Alcaligenes aquatilis* BU33N to other *Alcaligenes* species is presented in **Fig 2**. Further characterization of the genomic relatedness between BU33N and QD168 revealed that they share *in silico* DDH and OrthoANI similarities of 74.90 and 97.07%, respectively. The ANI reported here is slightly higher than the value (96.8%) reported by Durán et al. [19]. Although OrthoANI estimates have been reported to yield values that are 0.1 % higher than those obtained from ANI, the algorithm implemented in the former has been shown to provide a more robust means of estimating average nucleotide identity for taxonomic delineation [34]. Overall, these metrics revealed that the two strains belong to the same species and pending the availability of the genome of *Alcaligenes aquatilis* LMG 22996^T^, BU33N and QD168 could be considered to be affiliated to *Alcaligenes aquatilis* on the basis of single gene markers.

The ability of BU33N to grow on solid media in presence of different hydrocarbons as the sole energy and carbon source was used as an indicator of hydrocarbon degradation. BU33N grew successfully on a range of hydrocarbons including crude oil, phenanthrene, naphthalene, fluoranthene, pristane, pyrene, carbazole, octadecane and xylene, and but not on dibenzothiophen, dibenzofuran, squalene and anthracene. Recently, *A*. *aquatilis* QD168 was shown to be capable of utilizing several hydrocarbons such as benzoate, toluene, biphenyl and benzene as carbon sources [20]. The ability of *Alcaligenes faecalis* to utilize polyaromatic hydrocarbons [pyrene, chrysene and benzo(*a*)pyrene] as sole carbon sources and to degrade phenanthrene has been previously reported [40, 41].

**Fig 3:**
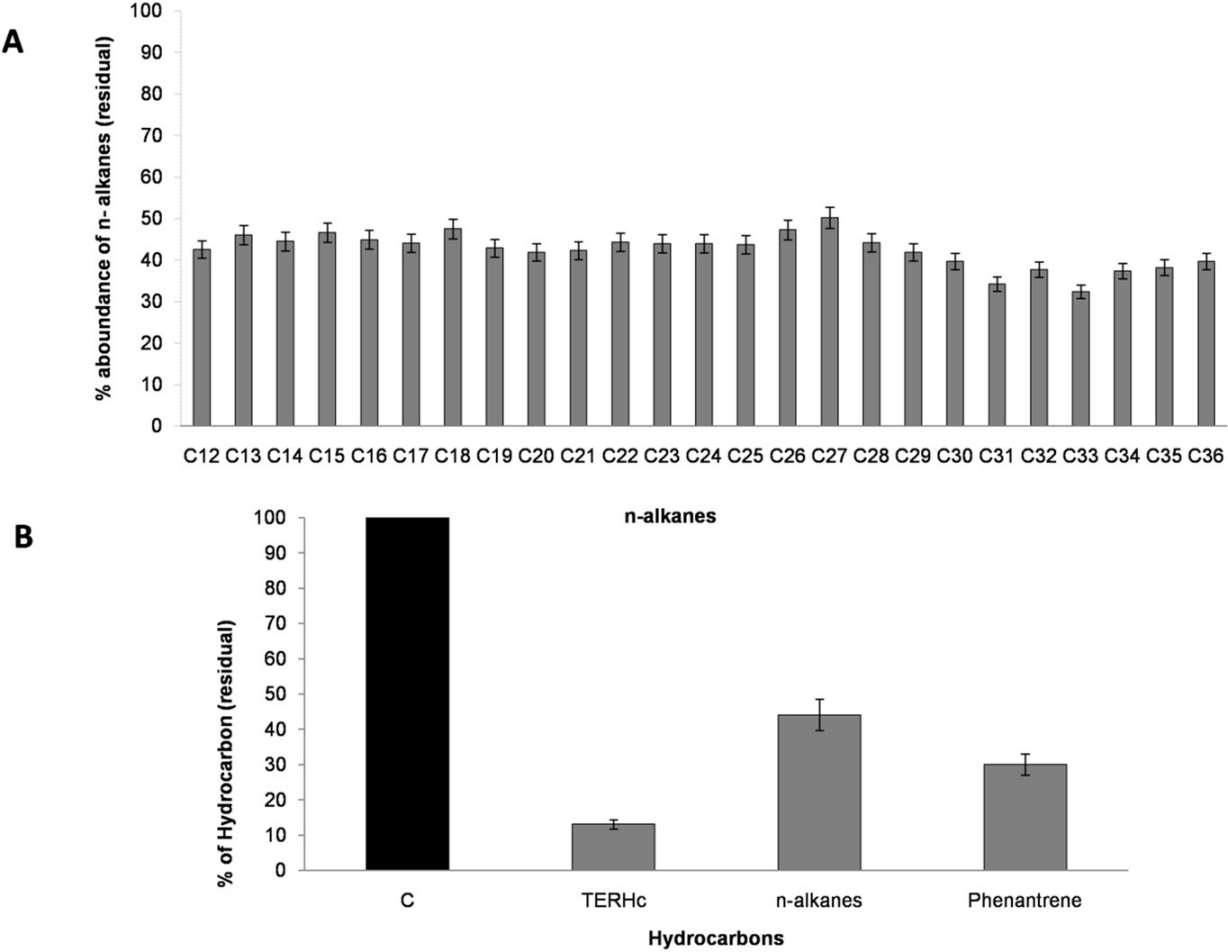
Degradation rate of hydrocarbon by BU33N. **A)** Relative percentage (%) of abundance (presence) of n-alkanes (C12—C_36_) after 21 days of incubation by BU33N; **B)** Degradation rate (%, residual hydrocarbons) of crude oil (TERCHs and n-alkanes) and phenanthrene by *Alcaligenes aquatilis* BU33N after 21 days of incubation.

Based on these results BU33N was cultured in mineral medium supplemented with crude oil and phenanthrene as sole carbon sources. After 21 days of incubation, GF-FID analysis of culture supernatants indicated ~86%, ~56% and ~70% degradation of TERHc, n -alkanes and phenanthrene, respectively (**Fig 3A and 3B)**.

### Insights into the genome of *Alcaligenes aquatilis* BU33N

**Fig 4:**
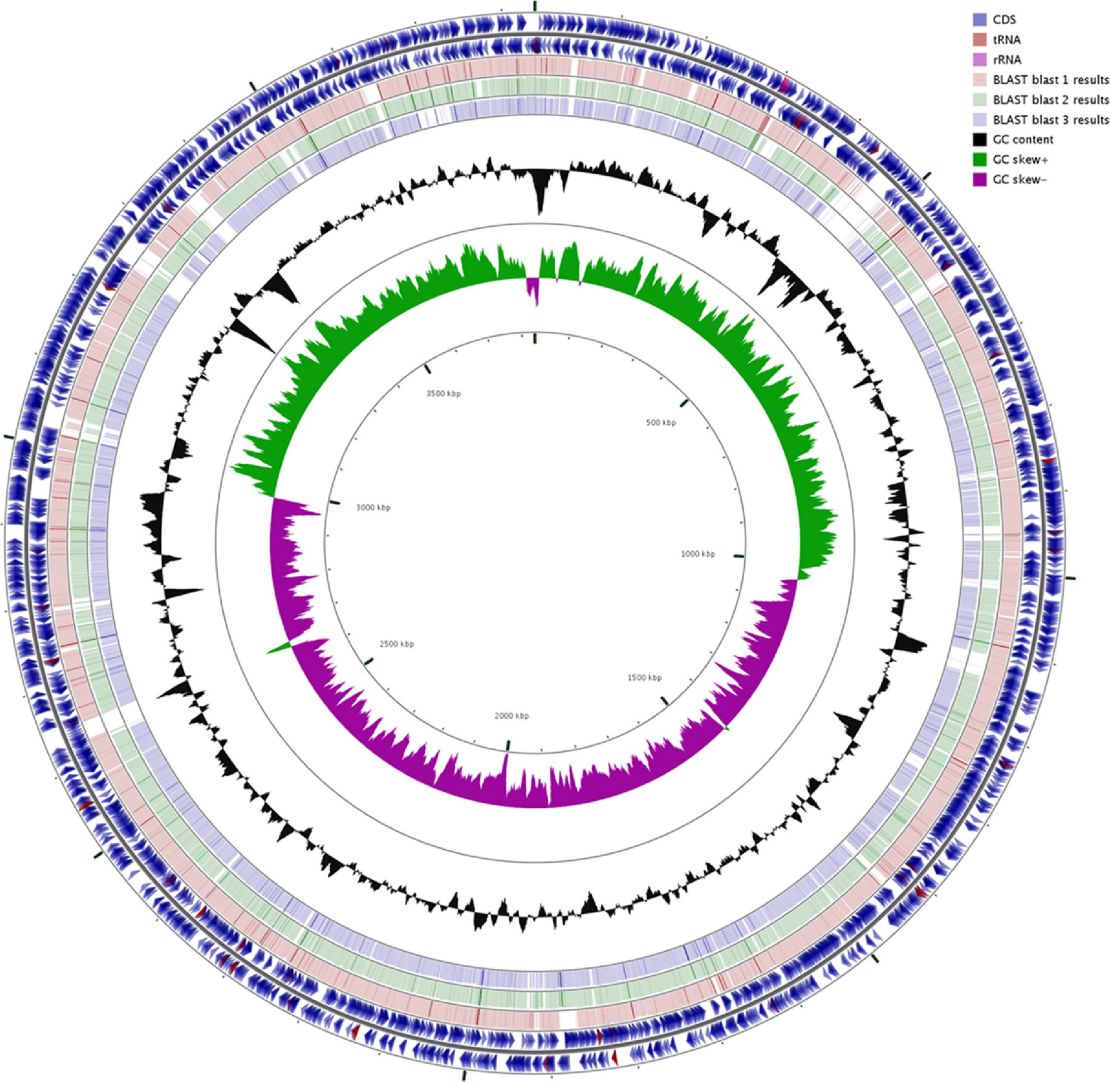
BLAST comparison of draft genome of *Alcaligenes aquatilis* against three *Alcaligenes* species, using GC View. The innermost rings depict GC content (Black) and GC Skew (purple/green) followed by concentric rings of query sequences colored according to BLAST identity. The outermost rings depict genomes of the following microbes *Alcaligenes aquatilis* QD168 (Pink), *Alcaligenes faecalis* (Green) and *Alcaligenes faecalis subsp*. *phenolicus (*Blue).

The *Alcaligenes aquatilis* BU33N genome was 3,838,299 bp in size, with a G+C content of 56.1 mol%, assembled as a single scaffold. Comparisons of the BU33N genome and three other members of the genus *Alcaligenes* showed similar genome sizes (≈4Mbp) (**Fig 4**). Genome annotation revealed that the BU33N genome encoded 3,506 protein-coding genes and 56 rRNA genes. Approximately 56% of the genes could be functionally assigned by the RAST subsystems.

Annotation of the BU33N genome showed the presence of several genes potentially involved in stress- adaptation. We identified multiple genes related to the osmotic stress (23 genes), oxidative stress (63 genes) and to heat and cold stress (21 genes) **(S2 Table**). Putative osmotic stress genes include ectoine biosynthesis and regulation genes, betaine biosynthesis genes and choline and betaine uptake genes. Oxidative stress genes were potentially involved in protection from Reactive Oxygen Species such as Superoxide dismutase, Manganese superoxide dismutase and Redox-sensitive transcriptional activator SoxR **(S2 Table**). Genes encoding cold shock elements (CspA family proteins) and heat shock elements (dnaK gene cluster) were identified.

**Fig 5:**
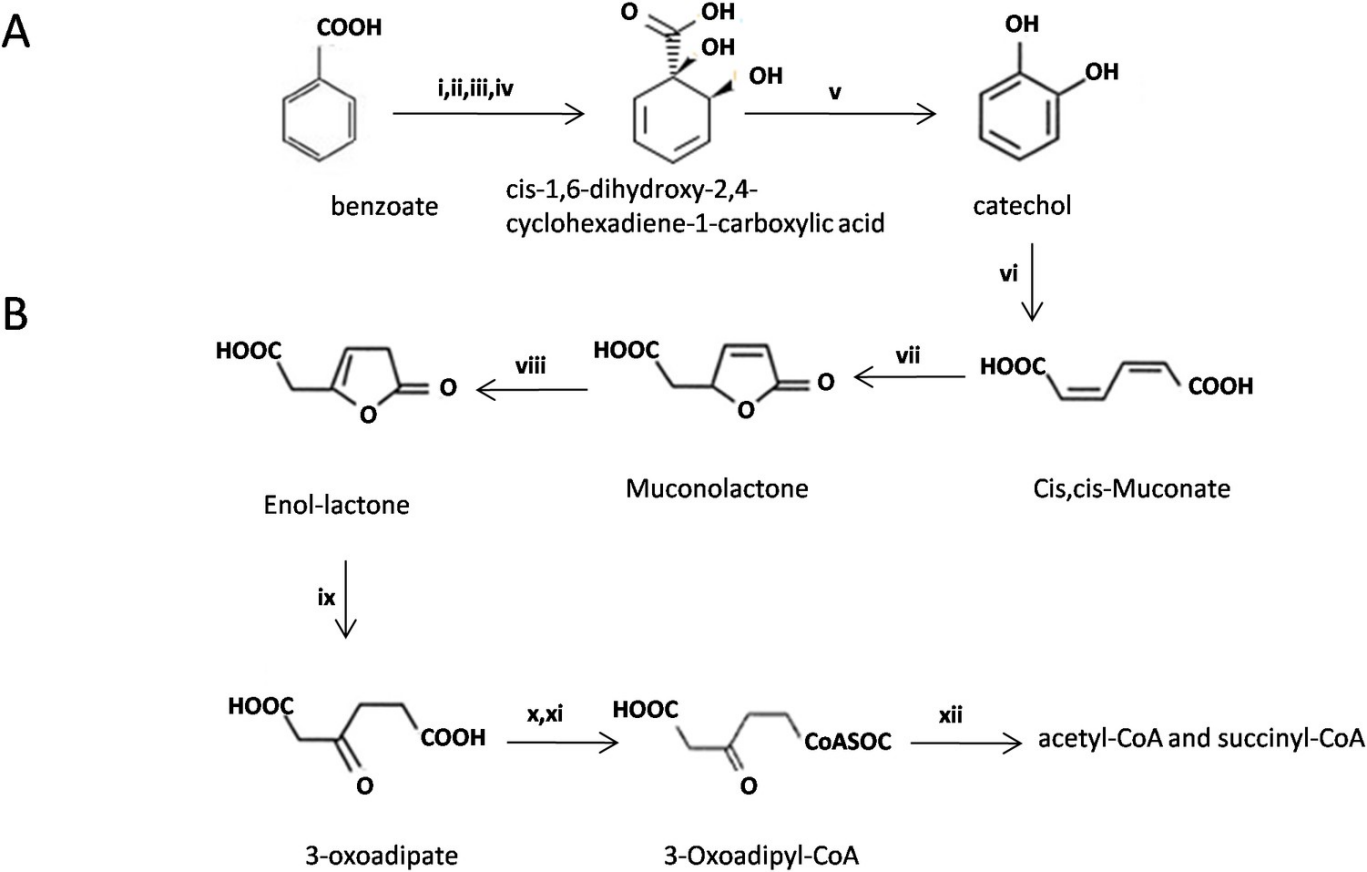
The benzoate (A) and catechol (B) biodegradation pathway in BU33N: A (i) benzoate 1, 2-dioxygenase alpha subunit (EC 1.14.12.10), (ii) beta subunit (EC 1.14.12.10), (iii) Ortho-halobenzoate 1, 2-dioxygenase alpha-ISP protein OhbA, (iv) beta-ISP protein OhbB (v) by 1, 2-dihydroxycyclohexa-3, 5-diene-1-carboxylate dehydrogenase (EC 1.3.1.25). B (vi) catechol 1, 2-dioxygenase (EC 1.13.11.1), (vii) muconatecycloisomerase (EC 5.5.1.1), (viii) muconolactone isomerase (EC 5.3.3.4), (ix) beta-ketoadipate enol-lactone hydrolase (EC 3.1.1.24), (x) 3-oxoadipate CoA-transferase subunit A (EC 2.8.3.6), (xi) 3-oxoadipate CoA-transferase subunit B (EC 2.8.3.6) and (xii) beta-ketoadipyl CoA thiolase (EC 2.3.1.-).

Based on the RAST annotation, 97 genes belonging to the category Metabolism of Aromatic Compounds were identified **(S3 Table**). The BU33N genome encodes a complete pathway for the aerobic degradation of benzoate [42], via the oxidation of benzoate to cis-1,6-dihydroxy-2,4-cyclohexadiene-1-carboxylic acid (DHC) and DHC to catechol (**Fig 5A**). This reaction is catalyzed by an enzyme complex that are encoded on two different gene clusters in the BU33N genome **(S3 Table**). Genes of protein (i), (ii) and (v) located in the first cluster (5,093 base pairs) along with three other genes, namely benzoate dioxygenase (ferredoxin reductase component) and two copies of benzoate MFS transporter (BenK).The second cluster (3,509 base pairs) contains four genes including those encoding proteins (iii) and (iv).

**Fig 6:**
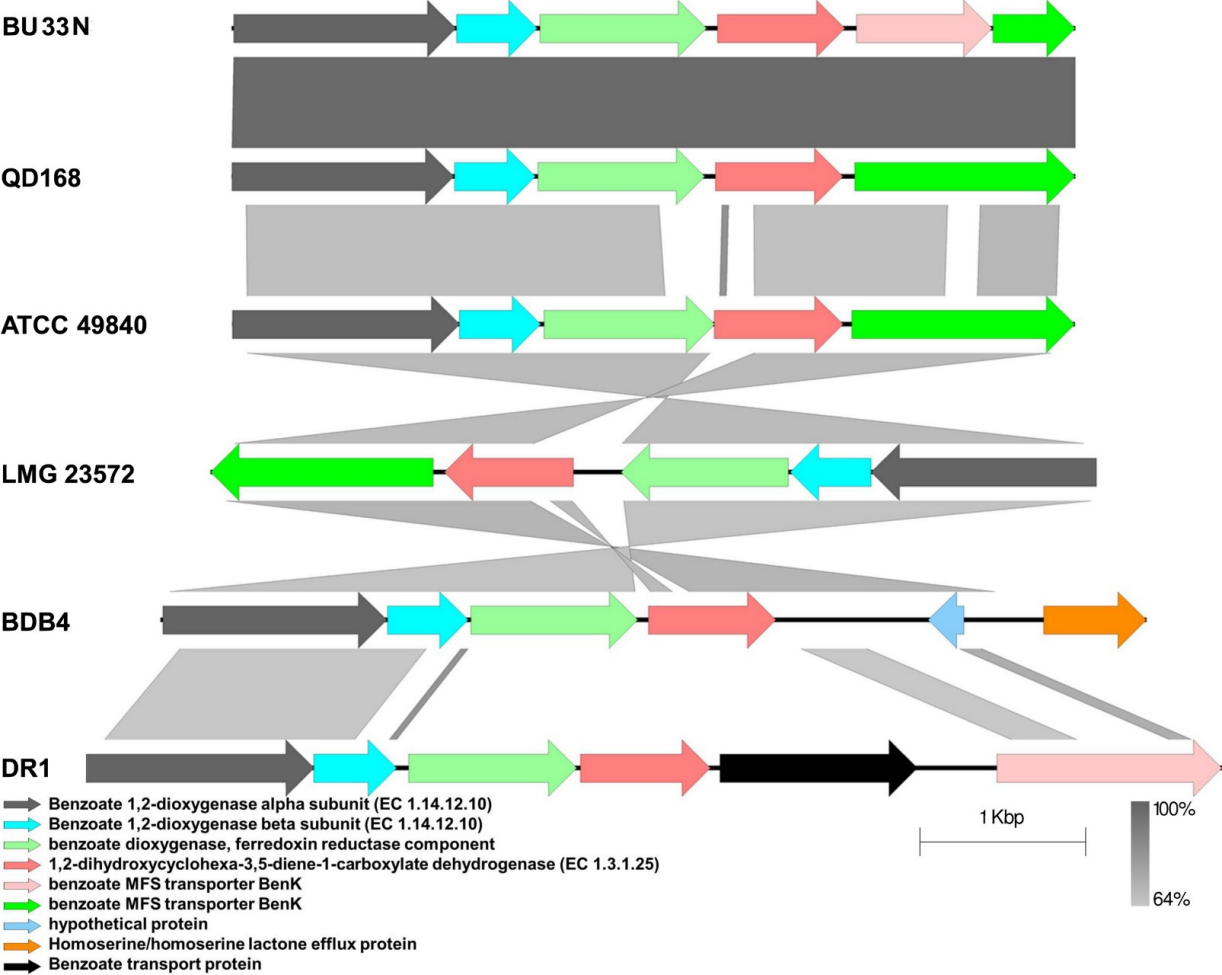
Gene organization of the first benzoate degradation enzymes encoding gene cluster in *Alcaligenes aquatilis* BU33N compared to that of selected hydrocarbon degrading bacteria. The strain identities, BU33N, QD168, BDB4, DR1, ATCC 49840 and LMG 23572 represent the bacteria *Alcaligenes aquatilis* BU33N, *Alcaligenes aquatilis* QD168, *Alcaligenes faecalis* BDB4, *Acinetobacter oleivorans* DR1, *Marinobacter hydrocarbonoclasticus* ATCC 49840 and *Pseudomonas xanthomarina* LMG 23572, respectively.

Comparison of the gene clusters in BU33N with those of selected hydrocarbon degrading bacteria (*Alcaligenes aquatilis* QD168, *Alcaligenes faecalis* BDB4, *Acinetobacter oleivorans* DR1, *Marinobacter hydrocarbonoclasticus* ATCC 49840 and *Pseudomonas xanthomarina* LMG 23572) showed that the genomic organization of the regions in which these proteins are encoded varied between organisms. However, the organization of genes encoding proteins (i), (ii) and (v) show high levels of synteny and conservation among the compared strains (**Fig 6**). However, sequence homology among the protein sequences encoded by these genes ranged between 64.00 to 99.74%. Higher level of orthology was observed between BU33N and its closest relative, QD168, with amino acid identity values ranging between 97.99 and 99.74%. On the other hand, the organization of the genomic locations of genes encoding iii and iv were different in all five organisms included in the analysis. The co-localization of genes associated with a common function is a well-known phenomenon in bacteria where these genes are often co- regulated [43]. On the other hand, the conservation of genes linked to specific function among different organisms could be an indication of common evolutionary history [44]. Consequently, the conservation and co-localization of major genes of aerobic degradation of benzoate may reflect a common origin of the genomic locus associated with the function.

**Fig 7:**
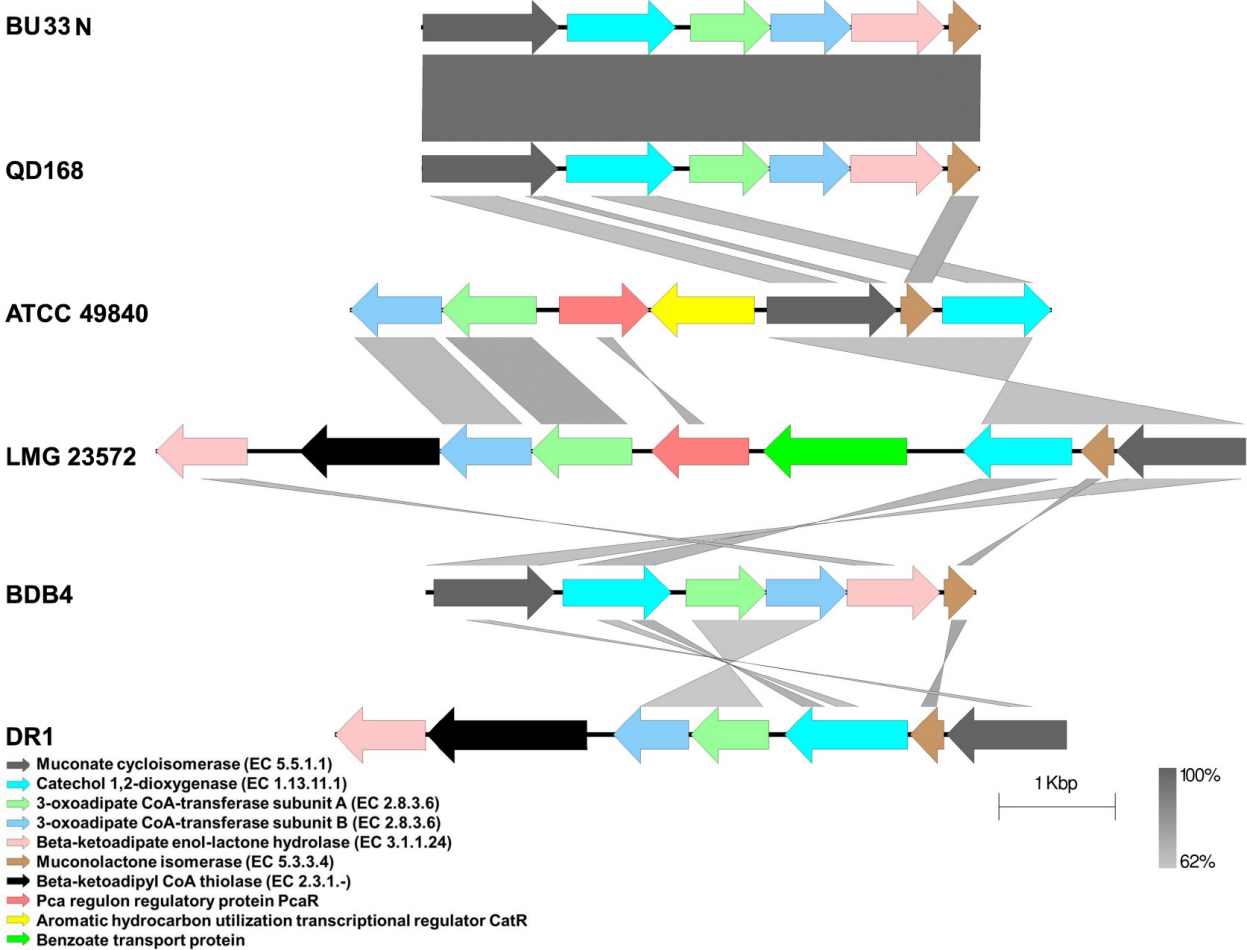
Gene organization of the first catechol degradation enzymes encoding gene cluster in *Alcaligenes aquatilis* BU33N compared to that of selected hydrocarbon degrading bacteria. The strain identities, BU33N, QD168, BDB4, DR1, ATCC 49840 and LMG 23572 represent the bacteria *Alcaligenes aquatilis* BU33N, *Alcaligenes*. *aquatilis* QD168, *Alcaligenes faecalis* BDB4, *Acinetobacter oleivorans* DR1, *Marinobacter hydrocarbonoclasticus* ATCC 49840 and *Pseudomonas xanthomarina* LMG 23572, respectively.

The BU33N genome encodes two loci encoding enzymes for the degradation of catechol to acetyl-CoA or succinyl-CoA (**Fig 5B**). The first locus encodes six enzymes (enzymes vi–xii) which catalyze the conversion of catechol to 3-Oxoadipyl-CoA. The final step involving the conversion of 3-Oxoadipyl-CoA to acetyl-CoA and succinyl-CoA is catalyzed by beta-ketoadipyl CoA thiolase (EC 2.3.1.-), encoded by a gene located 167,710 base pairs upstream of the first cluster in the genome of BU33N. Comparison of the BU33N catechol degradation gene clusters and those of *Alcaligenes aquatilis* QD168, *Alcaligenes faecalis* BDB4, *Acinetobacter oleivorans* DR1, *Marinobacter hydrocarbonoclasticus* ATCC 49840 and *Pseudomonas xanthomarina* LMG 23572 showed that these clusters are syntenous and conserved in BU33N, QD168 and BDB4 with beta-ketoadipyl CoA thiolase (EC 2.3.1) gene located upstream of the gene cluster encoding the rest of the enzymes. However, the gene which encodes this enzyme is produced in the same locus as the rest of the catechol degrading enzymes in Dr1 and LMG 2357 (**Fig 7**). Previous studies reported that these pathways play an essential role in aromatic compound degradation and it was found in various oil degrading bacteria such as *Pseudomonas*, *Franconibacter* and *Marinobacter* [42, 45, 46]. Genomic analysis of *A*. *aquatilis* QD168 also revealed a repertoire of genes linked to the catabolic pathways for aromatic compounds [20].

**Fig 8:**
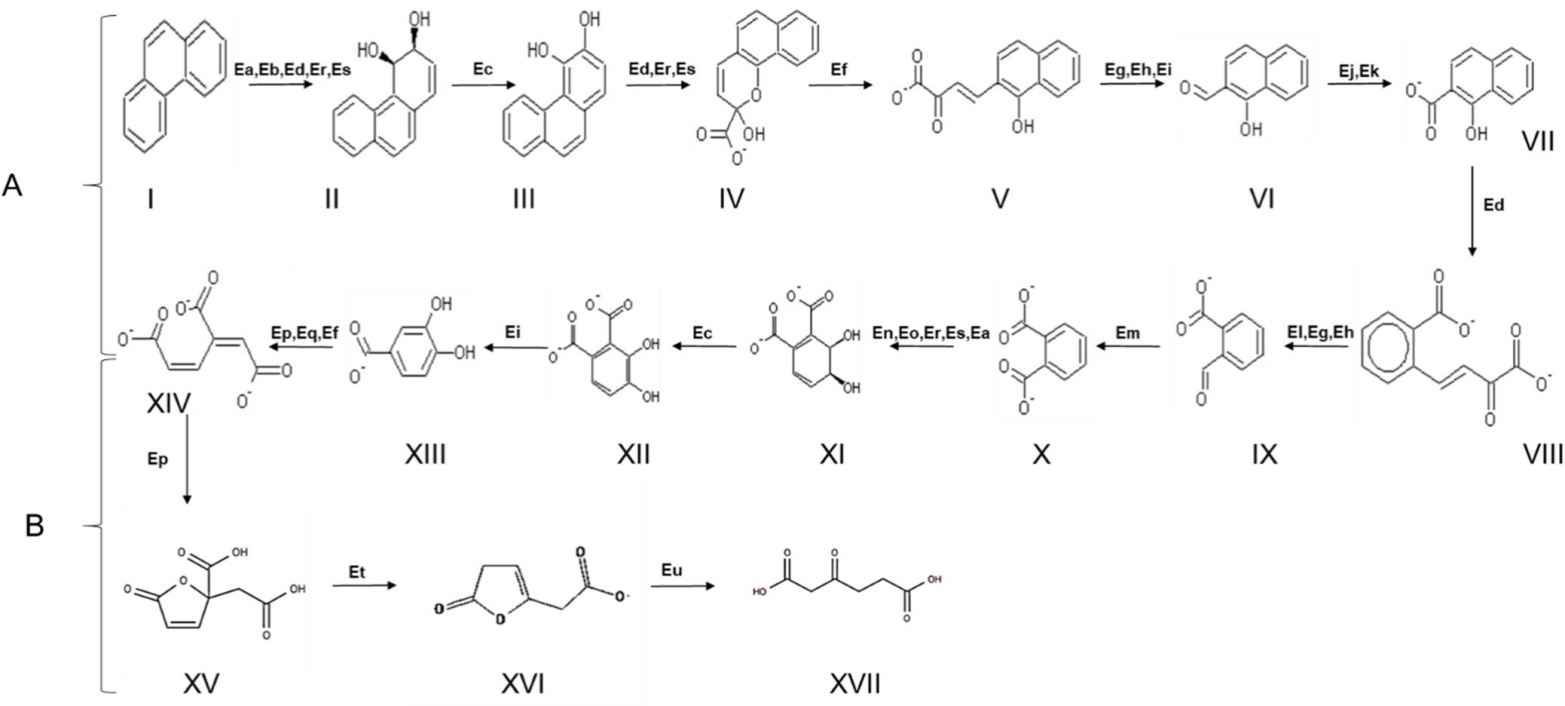
The A) phenanthrene degradation and B) β-ketoadipate pathways in *Alcaligenes aquatilis* BU33N. (I) Phenanthrene, (II) cis-3,4-Dihydroxy-3,4-dihydrophenanthrene, (III) 3,4-Dihydroxyphenanthrene, (IV) 2-Hydroxy-2H-benzo[h]chromene-2-carboxylate, (V) trans-4-(1'-Hydroxynaphth-2'-yl)-2-oxobut-3-enoate, (VI) 1-Hydroxy-2-naphthaldehyde, (VII) 1-Hydroxy-2-naphthoate, (VIII) trans-2'-Carboxybenzalpyruvate, (IX) 2-Carboxybenzaldehyde, (X) Phthalate, (XI) Phthalate 3,4-cis-dihydrodiol, (XII) 3,4-Dihydroxyphthalate, (XIII) 3,4-Dihydroxybenzoate, (XIV) 3-Carboxy-cis,cis-muconate, (XV) 4-carboxymuconolactone, (XVI) ß-ketoadipate enol-lactone and (XVII) 3-Oxoadipic acid; Beta-ketoadipate. Predicted enzymes include, (Ea) Ring hydroxylating dioxygenase, alpha subunit (EC 1.14.12.13), (Eb) 3-phenylpropionate dioxygenase beta subunit (EC 1.14.1.-), (Ec) 1,2-dihydroxycyclohexa-3,5-diene-1-carboxylate dehydrogenase (EC 1.3.1.25), (Ed) Biphenyl-2,3-diol 1,2-dioxygenase (EC 1.13.11.39), (Ef) Maleylacetoacetate isomerase (EC 5.2.1.2), (Eg) Possible carboxymuconolactone decarboxylase family protein (EC 4.1.1.44), (Eh) 4-hydroxy-2-oxovalerate aldolase (EC 4.1.3.39), (Ei) 4-carboxymuconolactone decarboxylase (EC 4.1.1.44), (Ej) Acetaldehyde dehydrogenase (EC 1.2.1.10), (Ek) Acetaldehyde dehydrogenase, acetylating, (EC 1.2.1.10), (El) carboxymuconolactone decarboxylase, (Em) Aldehyde dehydrogenase (EC 1.2.1.3), (En) Benzoate 1,2-dioxygenase beta subunit (EC 1.14.12.10), (Eo) Benzoate 1,2-dioxygenase alpha subunit (EC 1.14.12.10), (Ep) Muconatecycloisomerase (EC 5.5.1.1), (Eq) Homogentisate 1,2-dioxygenase (EC 1.13.11.5), (Er) Ortho-halobenzoate 1,2-dioxygenase beta-ISP protein OhbA, (Es) Ortho-halobenzoate 1,2-dioxygenase alpha-ISP protein OhbB, (Ep) Muconatecycloisomerase (EC 5.5.1.1), (Et) carboxymuconolactone decarboxylase and (Eu) Beta-ketoadipate enol-lactone hydrolase (EC 3.1.1.24).

To further interrogate the genomic basis for the ability of BU33N to utilize complex aromatic hydrocarbons, the phenanthrene biodegradation pathway was identified and reconstructed (**Fig 8**). On the basis of the identified genes, BU33N could be predicted to catabolize phenanthrene via the ortho-cleavage pathway [47, 48] to yield 3-carboxy-cis cis-muconate and subsequently beta-ketoadipate [49] through the β-ketoadipate pathway (**Fig 8**). Unlike the benzoate degradation pathway described above, the enzymes involved in phenanthrene biodegradation are encoded in different genomic regions and only genes for (EC 1.14.12.10: benzoate 1,2-dioxygenase), (EC 1.3.1.25:1,2-dihydroxycyclohexa-3,5-diene-1-carboxylate dehydrogenase), (EC 1.2.1.10: Acetaldehyde dehydrogenase) and (EC 4.1.3.39: 4-hydroxy-2-oxovalerate aldolase) were positioned within the major aromatic compound degradation gene clusters.

Interestingly, although hydrocarbon substrate screening of BU33N suggested the capacity to utilize n-alkanes, no known genes associated with alkane degradation (such as AlkB) were identified in the BU33N genome. This suggests that this organism uses a unidentified alkane degradation pathway.

Genome analysis confirmed the presence of multiple genes involved in the synthesis of biosurfactants, supporting the biosurfactant assay results. These include phosphomannomutase, dTDP-4-dehydrorhamnose 3,5-epimerase, Malonyl CoA-ACP transacylase and peptidoglycan glycosyltransferase (EC 2.4.1.129). These genes are present in well known biosurfactant-producing microorganisms such as *Pseudomonas putida* CSV86 [45].

Hydrocarbon contamination is also associated with heavy metals pollution. Bacteria harboring both heavy metals resistance genes and aromatic compound degradation traits would be highly interesting because often hydrocarbon pollution occurs concurrently with heavy metals contamination. The BU33N genome harbors heavy metals resistance genes which code for transcriptional regulator MerR family, cobalt-zinc-cadmium resistance protein CzcA and probable Co/Zn/Cd efflux system membrane fusion proteins. Similar set of genes were reported in the heavy metals- resistant bacterium *Pseudomonas putida* CSV86[45]. Additionally, arsenic resistance features are presents in BU33N genome and include genes encoding arsenate reductase (EC 1.20.4.1), arsenical-resistance protein ACR3 and arsenic resistance protein ArsH **(S4 Table)** [50].

## Conclusion

The isolation of strain *Alcaligenes aquatilis* BU33N and successive physiological and metabolic characterization combined with genomic analyses constitutes an interesting strategy to illustrate the bioremediation potential of this strain. The overall data will help the development of appropriate approach of autochthonous bioaugmentation in petroleum-polluted sites.

## Supporting information

S1 Table 16 rRNA genes similarity of BU33N strain (CP022390.1) and all type strains of the genus *Alcaligenes* using MAFFT. (PDF)

S2 Table Key enzymes involved in stress response in BU33N genome. (PDF)

S3 Table Key enzymes encoded in the *Alcaligenes aquatilis* BU33N genome associated with aromatic hydrocarbon degradation and biosurfactant synthesis from Rast and Eggnog annotation. (PDF)

S4 Table Heavy metals and drug resistance proteins encoded in BU33N genome. (PDF)

## References

[1] Jiang Z, Huang Y, Xu X, Liao Y, Shou L, Liu J, et al Advance in the toxic effects of petroleum water accommodated fraction on marine plankton. Acta Ecologica Sinica. 2010;30(1):8–15. 10.1016/j.chnaes.2009.12.002

[2] Niazy Z, Hassanshahian M, Ataei A. Isolation and characterization of diesel-degrading Pseudomonas strains from diesel-contaminated soils in Iran (Fars province). Pollution. 2016;2(1):67–75. 10.7508/PJ.2016.01.007

[3] Cappello S, Genovese M, Denaro R, Santisi S, Volta A, Bonsignore M, et al Quick stimulation of Alcanivorax sp. by bioemulsificant EPS2003 on microcosm oil spill simulation. Brazilian Journal of Microbiology. 2014;45(4):1317–23. 10.1590/s1517-83822014000400023 25763036 PMC4323305

[4] Crisafi F, Genovese M, Smedile F, Russo D, Catalfamo M, Yakimov M, et al Bioremediation technologies for polluted seawater sampled after an oil-spill in Taranto Gulf (Italy): A comparison of biostimulation, bioaugmentation and use of a washing agent in microcosm studies. Marine pollution bulletin. 2016;106(1–2):119–26. 10.1016/j.marpolbul.2016.03.017 26992747

[5] Genovese M, Crisafi F, Denaro R, Cappello S, Russo D, Calogero R, et al Effective bioremediation strategy for rapid in situ cleanup of anoxic marine sediments in mesocosm oil spill simulation. Frontiers in microbiology. 2014;5:162 10.3389/fmicb.2014.00162 24782850 PMC3995047

[6] Head IM, Jones DM, Röling WF. Marine microorganisms make a meal of oil. Nature Reviews Microbiology. 2006;4(3):173 10.1038/nrmicro1348 16489346

[7] Joo H-S, Hirai M, Shoda M. Piggery wastewater treatment using Alcaligenes faecalis strain No. 4 with heterotrophic nitrification and aerobic denitrification. Water Research. 2006;40(16):3029–36. 10.1016/j.watres.2006.06.021 16893560

[8] Ju S, Zheng J, Lin J, Geng C, Zhu L, Guan Z, et al The complete genome sequence of Alcaligenes faecalis ZD02, a novel potential bionematocide. Journal of biotechnology. 2016;218:73–4. 10.1016/j.jbiotec.2015.12.001 26656226

[9] Euzeby J. Subspecies Names Automatically Created by Rule 46. International Journal of Systematic and Evolutionary Microbiology. 1996;46(3):830–.

[10] Schroll G, Busse H-J, Parrer G, Rölleke S, Lubitz W, Denner EB. Alcaligenes faecalis subsp. parafaecalis subsp. nov., a Bacterium Accumulating Poly-β-hydroxybutyrate from Acetone-butanol Bioprocess Residues. Systematic and applied microbiology. 2001;24(1):37–43. 11403397 10.1078/0723-2020-00001

[11] Rehfuss M, Urban J. Alcaligenes faecalis subsp. phenolicus subsp. nov. a phenol-degrading, denitrifying bacterium isolated from a graywater bioprocessor. Systematic and applied microbiology. 2005;28(5):421–9. 10.1016/j.syapm.2005.03.003 16094869

[12] Van Trappen S, Tan T-L, Samyn E, Vandamme P. Alcaligenes aquatilis sp. nov., a novel bacterium from sediments of the Weser Estuary, Germany, and a salt marsh on Shem Creek in Charleston Harbor, USA. International journal of systematic and evolutionary microbiology. 2005;55(6):2571–5. 10.1099/ijs.0.63849-0 16280529

[13] Lu C-Y, Li Y-Q, Tian Y, Han M-X, Rao MPN, Li Y-R, et al Alcaligenes endophyticus sp. nov., isolated from roots of Ammodendron bifolium. International journal of systematic and evolutionary microbiology. 2017;67(4):939–43. 10.1099/ijsem.0.001719 27959788

[14] Abbas S, Ahmed I, Iida T, Lee Y-J, Busse H-J, Fujiwara T, et al A heavy-metal tolerant novel bacterium, Alcaligenes pakistanensis sp. nov., isolated from industrial effluent in Pakistan. Antonie Van Leeuwenhoek. 2015;108(4):859–70. 10.1007/s10482-015-0540-1 26238381

[15] Liu X, Huang D, Wu J, Yu C, Zhou R, Liu C, et al The genome sequence of Alcaligenes faecalis NBIB-017 contains genes with potentially high activities against Erwinia carotovora. Genome announcements. 2016;4(2):e00222–16. 10.1128/genomeA.00222-16 27056227 PMC4824260

[16] Nakano M, Niwa M, Nishimura N. Development of a PCR-based method for monitoring the status of Alcaligenes species in the agricultural environment. Biocontrol science. 2014;19(1):23–31. 10.4265/bio.19.23 24670615

[17] Pan X, Lin D, Zheng Y, Zhang Q, Yin Y, Cai L, et al Biodegradation of DDT by Stenotrophomonas sp. DDT-1: characterization and genome functional analysis. Scientific reports. 2016;6:21332 10.1038/srep21332 26888254 PMC4758049

[18] Magthalin CJ, Varadharajan A, Swarnalatha S, Sekaran G. Cationic dispersant immobilized matrix for sequestering Cr (III) from contaminated soil. Materials Today: Proceedings. 2016;3(10):3697–702. 10.1016/j.matpr.2016.11.015

[19] Durán RE, Barra-Sanhueza B, Salvà-Serra F, Méndez V, Jaén-Luchoro D, Moore ERB, et al Complete Genome Sequence of the Marine Hydrocarbon Degrader Alcaligenes aquatilis QD168, Isolated from Crude Oil-Polluted Sediment of Quintero Bay, Central Chile. Microbiology Resource Announcements. 2019;8(5):e01664–18. 10.1128/MRA.01664-18 30714040 PMC6357646

[20] Durán RE, Méndez V, Rodríguez-Castro L, Barra-Sanhueza B, Salvà-Serra F, Moore ER, et al Genomic and Physiological Traits of the Marine Bacterium Alcaligenes aquatilis QD168 Isolated From Quintero Bay, Central Chile, Reveal a Robust Adaptive Response to Environmental Stressors. Frontiers in microbiology. 2019;10:528 10.3389/fmicb.2019.00528 31024465 PMC6460240

[21] Santisi S, Cappello S, Catalfamo M, Mancini G, Hassanshahian M, Genovese L, et al Biodegradation of crude oil by individual bacterial strains and a mixed bacterial consortium. Brazilian Journal of Microbiology. 2015;46(2):377–87. 10.1590/S1517-838246120131276 26273252 PMC4507529

[22] Hassanshahian M, Emtiazi G, Caruso G, Cappello S. Bioremediation (bioaugmentation/biostimulation) trials of oil polluted seawater: a mesocosm simulation study. Marine environmental research. 2014;95:28–38. 10.1016/j.marenvres.2013.12.010 24388285

[23] Pinzon NM, Ju L-K. Analysis of rhamnolipid biosurfactants by methylene blue complexation. Applied microbiology and biotechnology. 2009;82(5):975–81. 10.1007/s00253-009-1896-9 19214498

[24] Youssef NH, Duncan KE, Nagle DP, Savage KN, Knapp RM, McInerney MJ. Comparison of methods to detect biosurfactant production by diverse microorganisms. Journal of microbiological methods. 2004;56(3):339–47. 10.1016/j.mimet.2003.11.001 14967225

[25] Mahjoubi M, Jaouani A, Guesmi A, Amor SB, Jouini A, Cherif H, et al Hydrocarbonoclastic bacteria isolated from petroleum contaminated sites in Tunisia: isolation, identification and characterization of the biotechnological potential. New biotechnology. 2013;30(6):723–33. 10.1016/j.nbt.2013.03.004 23541698

[26] Stanton S, Meyer JJM, Van der Merwe CF. An evaluation of the endophytic colonies present in pathogenic and non-pathogenic Vanguerieae using electron microscopy. South African journal of botany. 2013;86:41–5. 10.1016/j.sajb.2013.01.007

[27] Kalman S, Kiehne KL, Libs JL, Yamamoto T. Cloning of a novel cryIC-type gene from a strain of Bacillus thuringiensis subsp. galleriae. Appl Environ Microbiol. 1993;59(4):1131–7. 8476286 10.1128/aem.59.4.1131-1137.1993 PMC202250

[28] Bankevich A, Nurk S, Antipov D, Gurevich AA, Dvorkin M, Kulikov AS, et al SPAdes: a new genome assembly algorithm and its applications to single-cell sequencing. Journal of computational biology. 2012;19(5):455–77. 10.1089/cmb.2012.0021 22506599 PMC3342519

[29] Lagesen K, Hallin P, Rødland EA, Stærfeldt H-H, Rognes T, Ussery DW. RNAmmer: consistent and rapid annotation of ribosomal RNA genes. Nucleic Acids Res. 2007;35(9):3100–8. 10.1093/nar/gkm160 17452365 PMC1888812

[30] Yoon S-H, Ha S-M, Kwon S, Lim J, Kim Y, Seo H, et al Introducing EzBioCloud: a taxonomically united database of 16S rRNA gene sequences and whole-genome assemblies. International journal of systematic and evolutionary microbiology. 2017;67(5):1613–7. 10.1099/ijsem.0.001755 28005526 PMC5563544

[31] Katoh K, Standley DM. MAFFT: iterative refinement and additional methods Multiple Sequence Alignment Methods: Springer; 2014 p. 131–46. 10.1007/978-1-62703-646-7_8 24170399

[32] Silla-Martínez JM, Capella-Gutiérrez S, Gabaldón T. trimAl: a tool for automated alignment trimming in large-scale phylogenetic analyses. Bioinformatics. 2009;25(15):1972–3. 10.1093/bioinformatics/btp348 19505945 PMC2712344

[33] Meier-Kolthoff JP, Auch AF, Klenk H-P, Göker M. Genome sequence-based species delimitation with confidence intervals and improved distance functions. BMC Bioinformatics. 2013;14(1):1–14. 10.1186/1471-2105-14-60 23432962 PMC3665452

[34] Lee I, Ouk Kim Y, Park S-C, Chun J. OrthoANI: An improved algorithm and software for calculating average nucleotide identity. Int J Syst Evol Microbiol. 2016;66(2):1100–3. 10.1099/ijsem.0.000760 26585518

[35] Aziz RK, Bartels D, Best AA, DeJongh M, Disz T, Edwards RA, et al The RAST Server: rapid annotations using subsystems technology. BMC genomics. 2008;9(1):75 10.1186/1471-2164-9-75 18261238 PMC2265698

[36] Grant JR, Stothard P. The CGView Server: a comparative genomics tool for circular genomes. Nucleic acids research. 2008;36(suppl_2):W181–W4. 10.1093/nar/gkn179 18411202 PMC2447734

[37] Seemann T. Prokka: rapid prokaryotic genome annotation. Bioinformatics. 2014;30(14):2068–9. 10.1093/bioinformatics/btu153 24642063

[38] Afgan E, Baker D, Van den Beek M, Blankenberg D, Bouvier D, Čech M, et al The Galaxy platform for accessible, reproducible and collaborative biomedical analyses: 2016 update. Nucleic acids research. 2016;44(W1):W3–W10. 10.1093/nar/gkw343 27137889 PMC4987906

[39] Sullivan MJ, Petty NK, Beatson SA. Easyfig: a genome comparison visualizer. Bioinformatics. 2011;27(7):1009–10. 10.1093/bioinformatics/btr039 21278367 PMC3065679

[40] Abd-Elsalam HE, Hafez EE, Hussain AA, Ali AG, El-Hanafy AA. Isolation and identification of three-rings polyaromatic hydrocarbons (anthracene and phenanthrene) degrading bacteria. Am Eurasian J Agric Environ Sci. 2009;5:31–8.

[41] Singha LP, Kotoky R, Pandey P. Draft Genome Sequence of Alcaligenes faecalis BDB4, a Polyaromatic Hydrocarbon-Degrading Bacterium Isolated from Crude Oil-Contaminated Soil. Genome announcements. 2017;5(48):e01346–17. 10.1128/genomeA.01346-17 29192081 PMC5722067

[42] Pal S, Kundu A, Banerjee TD, Mohapatra B, Roy A, Manna R, et al Genome analysis of crude oil degrading Franconibacter pulveris strain DJ34 revealed its genetic basis for hydrocarbon degradation and survival in oil contaminated environment. Genomics. 2017;109(5–6):374–82. 10.1016/j.ygeno.2017.06.002 28625866

[43] Rocha EP, Cornet E, Michel B. Comparative and evolutionary analysis of the bacterial homologous recombination systems. PLoS genetics. 2005;1(2):e15 10.1371/journal.pgen.0010015 16132081 PMC1193525

[44] Almagro G, Viale AM, Montero M, Rahimpour M, Muñoz FJ, Baroja-Fernández E, et al Comparative Genomic and Phylogenetic Analyses of Gammaproteobacterial glg Genes Traced the Origin of the Escherichia coli Glycogen glgBXCAP Operon to the Last Common Ancestor of the Sister Orders Enterobacteriales and Pasteurellales. PLOS ONE. 2015;10(1):e0115516 10.1371/journal.pone.0115516 25607991 PMC4301808

[45] Paliwal V, Raju SC, Modak A, Phale PS, Purohit HJ. Pseudomonas putida CSV86: a candidate genome for genetic bioaugmentation. PLoS One. 2014;9(1):e84000 10.1371/journal.pone.0084000 24475028 PMC3901652

[46] Rani S, Jeon WJ, Koh H-W, Kim Y-E, Kang M-S, Park S-J. Genomic potential of Marinobacter salinus Hb8 T as sulfur oxidizing and aromatic hydrocarbon degrading bacterium. Marine Genomics. 2017;34:19–21. 10.1016/j.margen.2017.02.005

[47] Macchi M, Martinez M, Tauil RN, Valacco M, Morelli I, Coppotelli B. Insights into the genome and proteome of Sphingomonas paucimobilis strain 20006FA involved in the regulation of polycyclic aromatic hydrocarbon degradation. World Journal of Microbiology and Biotechnology. 2018;34(1):7 10.1007/s11274-017-2391-6 29214360

[48] Seo J-S, Keum Y-S, Li QX. Bacterial degradation of aromatic compounds. International journal of environmental research and public health. 2009;6(1):278–309. 10.3390/ijerph6010278 19440284 PMC2672333

[49] Yamanashi T, Kim S-Y, Hara H, Funa N. In vitro reconstitution of the catabolic reactions catalyzed by PcaHG, PcaB, and PcaL: the protocatechuate branch of the β-ketoadipate pathway in Rhodococcus jostii RHA1. Bioscience, biotechnology, and biochemistry. 2015;79(5):830–5. 10.1080/09168451.2014.993915 25558786

[50] Serrano AE, Escudero LV, Tebes-Cayo C, Acosta M, Encalada O, Fernández-Moroso S, et al First draft genome sequence of a strain from the genus Fusibacter isolated from Salar de Ascotán in Northern Chile. Standards in genomic sciences. 2017;12(1):43 10.1186/s40793-017-0252-4 28770028 PMC5525254

