# Supplementary material for: The genome of Alcaligenes aquatilis strain BU33N: Insights into hydrocarbon degradation capacity
Source: PLoS One. 2019 Sep 24;14(9):e0221574. doi: 10.1371/journal.pone.0221574 (PMC6759156; doi:10.1371/journal.pone.0221574)
Supplement: S1 Table — (PDF) [file pone.0221574.s001.pdf]

S1 Table. 16 rRNA genes similarity of BUN33 strain (CP022390.1) and all type strains of the genus *Alcaligenes* using MAFFT

Type strain	AB920828.1	AJ242986.1	AJ937889.1	CP022390.1	CP032153.2	CP032153.1	CP032153.3	KR967368.1	D88008.1	AY296718.1	U96927.1
**AB920828.1**	100	98.71	98.08	98.65	98.65	98.79	98.65	97.16	98.15	96.88	88.52
**AJ242986.1**	98.71	100	99.71	99.79	99.79	99.65	99.5	98.3	98.65	97.31	89.05
**AJ937889.1**	98.08	99.71	100	99.91	99.91	99.72	99.54	97.97	98.5	97.19	87.39
**CP022390.1**	98.65	99.79	99.91	100	100	99.87	99.74	98.36	98.84	96.84	89.54
**CP032153.2**	98.65	99.79	99.91	100	100	99.87	99.74	98.36	98.84	96.84	89.54
**CP032153.1**	98.79	99.65	99.72	99.87	99.87	100	99.87	98.23	98.71	96.71	89.67
**CP032153.3**	98.65	99.5	99.54	99.74	99.74	99.87	100	98.1	98.57	96.57	89.81
**KR967368.1**	97.16	98.3	97.97	98.36	98.36	98.23	98.1	100	97.41	95.56	88.87
**D88008.1**	98.15	98.65	98.5	98.84	98.84	98.71	98.57	97.41	100	97.34	88.75
**AY296718.1**	96.88	97.31	97.19	96.84	96.84	96.71	96.57	95.56	97.34	100	87.11
**U96927.1**	88.52	89.05	87.39	89.54	89.54	89.67	89.81	88.87	88.75	87.11	100
