# Supplementary material for: The genome of Alcaligenes aquatilis strain BU33N: Insights into hydrocarbon degradation capacity
Source: PLoS One. 2019 Sep 24;14(9):e0221574. doi: 10.1371/journal.pone.0221574 (PMC6759156; doi:10.1371/journal.pone.0221574)
Supplement: S2 Table — (PDF) [file pone.0221574.s002.pdf]

S2Table. Key enzymes involved in stress response in BUN33 genome

Subsystem	Functional Roles
**Osmotic stress**	Osmotically inducible lipoprotein B precursor
	Osmotically inducible protein OsmY
	Aquaporin Z
	Outer membrane protein A precursor
	Ectoine hydroxylase (EC 1.17.-.-)
	L-ectoine synthase (EC 4.2.1.-)
	Diaminobutyrate-pyruvate aminotransferase (EC 2.6.1.46)
	Putative regulatory protein associated with the ectoine operon
	L-2,4-diaminobutyric acid acetyltransferase (EC 2.3.1.-)
	L-proline glycine betaine ABC transport system permease protein ProV (TC 3.A.1.12.1)
	L-proline glycine betaine ABC transport system permease protein ProW (TC 3.A.1.12.1)
	L-proline glycine betaine binding ABC transporter protein ProX (TC 3.A.1.12.1)
	High-affinity choline uptake protein BetT
	GbcA Glycine betaine demethylase subunit A
**Oxidative stress**	Superoxide dismutase [Fe] (EC 1.15.1.1)
	Redox-sensitive transcriptional activator SoxR
	Manganese superoxide dismutase (EC 1.15.1.1)
	transcriptional regulator, Crp/Fnr family
	Ferric uptake regulation protein FUR
	Redox-sensitive transcriptional activator SoxR
	Nitrite-sensitive transcriptional repressor NsrR
**Cold shock**	Cold shock protein CspE

	Cold shock protein CspA
	Cold shock protein CspD
	Cold shock protein CspE
**Heat shock**	Probable Fe(2+)-trafficking protein YggX
	Ribosomal protein L11 methyltransferase (EC 2.1.1.-)
	Nucleoside 5-triphosphatase RdgB (dHAPTP, dITP, XTP-specific) (EC 3.6.1.15)
	Ribonuclease PH (EC 2.7.7.56)
