# Supplementary material for: The genome of Alcaligenes aquatilis strain BU33N: Insights into hydrocarbon degradation capacity
Source: PLoS One. 2019 Sep 24;14(9):e0221574. doi: 10.1371/journal.pone.0221574 (PMC6759156; doi:10.1371/journal.pone.0221574)
Supplement: S3 Table — (PDF) [file pone.0221574.s003.pdf]

S3 Table. Key enzymes encoded in the *Alcaligenes aquatilis* BU33N genome associated with aromatic hydrocarbon degradation and biosurfactant synthesis from Rast and Eggnog annotation.

**Pathway**	**Protein/Enzyme**	**EC number**
**Benzoate degradation**	benzoate 1, 2-dioxygenase alpha subunit	EC 1.14.12.10
	benzoate 1, 2-dioxygenase beta subunit	EC 1.14.12.10
	Ortho-halobenzoate 1, 2-dioxygenase OhbA & OhbB	
	1, 2-dihydroxycyclohexa-3, 5-diene-1-carboxylate dehydrogenase	EC 1.3.1.25
	4-hydroxybenzoate transporter	
	P-hydroxybenzoate hydroxylase	EC 1.14.13.2
	Ring hydroxylating dioxygenase, alpha subunit	EC 1.14.12.13
	4-carboxymuconolactone decarboxylase	EC 4.1.1.44
	3-polyprenyl-4-hydroxybenzoate carboxy-lyase UbiX	EC 4.1.1.-
	3-polyprenyl-4-hydroxybenzoate carboxy-lyase	EC 4.1.1.-
	4-hydroxybenzoate polyprenyltransferase	EC 2.5.1.39
	4-carboxymuconolactone decarboxylase	EC 4.1.1.44
	Glutaryl-CoA dehydrogenase	EC 1.3.99.7
	Protocatechuate 4,5-dioxygenase	EC:1.13.11.3
	protocatechuate 4,5-dioxygenase subunit alpha	EC:1.13.11.3

	enoyl-CoA hydratase	EC:4.2.1.17
**Catechol**	catechol 1, 2-dioxygenase	EC 1.13.11.1
	muconatecycloisomerase	EC 5.5.1.1
	muconolactone isomerase	EC 5.3.3.4
	beta-ketoadipate enol-lactone hydrolase	EC 3.1.1.24
	3-oxoadipate CoA-transferase subunit A	EC 2.8.3.6
	3-oxoadipate CoA-transferase subunit B	EC 2.8.3.6
	beta-ketoadipyl CoA thiolase	EC 2.3.1.-
**Phenanthrene/Ketoadipate**	Ring hydroxylating dioxygenase, alpha subunit	EC 1.14.12.13
	3-phenylpropionate dioxygenase beta subunit	EC 1.14.1.-
	1,2-dihydroxycyclohexa-3,5-diene-1-carboxylate dehydrogenase	EC 1.3.1.25
	Biphenyl-2,3-diol 1,2-dioxygenase	EC 1.13.11.39
	Maleylacetoacetate isomerase	EC 5.2.1.2
	Possible carboxymuconolactone decarboxylase family protein	EC 4.1.1.44
	4-hydroxy-2-oxovalerate aldolase	EC 4.1.3.39
	4-carboxymuconolactone decarboxylase	EC 4.1.1.44
	Acetaldehyde dehydrogenase	(EC 1.2.1.10)
	Acetaldehyde dehydrogenase, acetylating,	EC 1.2.1.10

	carboxymuconolactone decarboxylase	
	Aldehyde dehydrogenase	EC 1.2.1.3
	Benzoate 1,2-dioxygenase beta subunit	EC 1.14.12.10
	Benzoate 1,2-dioxygenase alpha subunit	EC 1.14.12.10
	Muconate cycloisomerase	EC 5.5.1.1
	Homogentisate 1,2-dioxygenase	EC 1.13.11.5
	Ortho-halobenzoate 1,2-dioxygenase OhbA	
	Ortho-halobenzoate 1,2-dioxygenase OhbB	
	Muconate cycloisomerase	EC 5.5.1.1
	carboxymuconolactone decarboxylase	
	Beta-ketoadipate enol-lactone hydrolase	EC 3.1.1.24
**Dioxin degradation**	Acetaldehyde dehydrogenase	EC 1.2.1.10
	2-keto-4-pentenoate hydratase	EC 4.2.1.80
	Alcohol dehydrogenase	EC 1.1.1.1
	Biphenyl-2,3-diol 1,2-dioxygenase	EC 1.13.11.39
	2-hydroxy-6-oxo-6-phenylhexa-2,4-dienoate hydrolase	EC 3.7.1
	2-keto-4-pentenoate hydratase	EC 4.2.1.80
	Acetaldehyde dehydrogenase, acetylating	EC 1.2.1.10
	4-hydroxy-2-oxovalerate aldolase	EC 4.1.3.39

	2-keto-4-pentenoate hydratase	EC 4.2.1.80
	Salicylate hydroxylase	EC 1.14.13.1
	4-hydroxy-2-oxovalerate aldolase	EC4.1.3.39
**Salicylate and gentisate catabolism**	Salicylate hydroxylase	EC 1.14.13.1
	Fumarylacetoacetase	EC 3.7.1.2
	Maleate cis-trans isomerase	EC 5.2.1.1
	Gentisate 1,2-dioxygenase	EC 1.13.11.4
	salicylate esterase	
	Homogentisate 1,2-dioxygenase	EC 1.13.11.5
	Fumarylacetoacetase	EC 3.7.1.2
**Phenol hydroxylase,**	Phenol hydroxylase, assembly protein DmpK	
	Phenol hydroxylase, P1 oxygenase component DmpL	EC 1.14.13.7
	Phenol hydroxylase, P2 regulatory component DmpM	EC 1.14.13.7
	Phenol hydroxylase, FAD- and [2Fe-2S]	
	Phenol hydroxylase, P3 oxygenase component DmpN	EC 1.14.13.7
	Phenol hydroxylase, P4 oxygenase component DmpO	EC 1.14.13.7
**Biosurfactant synthesis**	dTDP-4-dehydrorhamnose 3,5-epimerase	EC 5.1.3.13
	3-oxoacyl-[acyl-carrier protein] reductase	EC 1.1.1.100
	Malonyl CoA-ACP transacylase	EC 2.3.1.39

peptidoglycan glycosyltransferase	EC 2.4.1.129
Phosphomannomutase	EC 5.4.2.8
Aspartokinase	EC 5.2.1.1
