# Supplementary material for: The genome of Alcaligenes aquatilis strain BU33N: Insights into hydrocarbon degradation capacity
Source: PLoS One. 2019 Sep 24;14(9):e0221574. doi: 10.1371/journal.pone.0221574 (PMC6759156; doi:10.1371/journal.pone.0221574)
Supplement: S4 Table — (PDF) [file pone.0221574.s004.pdf]

S4 Table. Heavy metals and drug resistance proteins encoded in BU33N genome

Function	Protein
Cobalt-zinc-cadmium resistance	
Cobalt-zinc-cadmium resistance protein	CzcD
Cobalt-zinc-cadmium resistance protein	CzcA
Putative copper efflux system protein	CusB
Probable Co/Zn/Cd efflux system	CzsB
Cobalt-zinc-cadmium efflux RND transporter, membrane fusion protein	CzcB
efflux system protein	CusA
DNA-binding heavy metal response regulator	CzrB
Heavy metal sensor histidine kinase	HMHK
Copper-sensing two-component system response regulator	CusR
Copper sensory histidine kinase	CusS
TRCd Cd(II)/Pb(II)-responsive transcriptional regulator	TRCd
Heavy metal resistance transcriptional regulator	HmrR
Nickel-cobalt-cadmium resistance	NccA
Arsenic resistance	
Arsenic efflux pump protein	arsB
Arsenical-resistance protein	ACR3
Resistance protein	ArsH
Arsenate reductase	Arsc
Copper tolerance	
Periplasmic divalent cation tolerance protein	CutA
Apolipoprotein N-acyltransferase / Copper homeostasis protein	CutE

cobalt efflux protein	CorC
Drug Resistance	
RND efflux system, outer membrane lipoprotein	CmeC
RND efflux system, inner membrane transporter	CmeB
RND efflux system, membrane fusion protein	CmeA
Multidrug and toxin extrusion (MATE) family efflux pump YdhE/NorM	
RND multidrug efflux transporter; Acriflavin resistance protein	
Macrolide export ATP-binding/permease protein (EC 3.6.3.-)	MacB
Macrolide-specific efflux protein	MacA
Type I secretion outer membrane protein, precursor	TolC
RND efflux system, outer membrane lipoprotein, NodT family	NodT
RND multidrug efflux transporter; Acriflavin resistance protein	
Multi antimicrobial extrusion protein (Na(+)/drug antiporter), MATE family of MDR efflux pumps	
